# Assessing the Role of Infant and Toddler MenACWY Immunisation in the UK: Does the Adolescent MenACWY Programme Provide Sufficient Protection?

**DOI:** 10.3390/vaccines11050940

**Published:** 2023-05-04

**Authors:** Katharina Schley, Jack C. Kowalik, Shannon M. Sullivan, Andrew Vyse, Carole Czudek, Eszter Tichy, Jamie Findlow

**Affiliations:** 1 Pfizer Pharma GmbH, Linkstraße 10, 10785 Berlin, Germany; 2 Pfizer Ltd., Walton Oaks, Dorking Rd., Tadworth KT20 7NS, UK; 3 Evidera/PPD, 27-35, rue Victor Hugo, 94853 Ivry-sur-Seine CEDEX, France; 4 Evidera/PPD, Bocskai ut 134-144, Dorottya Udvar, Building E, Floor 2, H-1113 Budapest, Hungary

**Keywords:** invasive meningococcal disease, vaccination, public health, health policy, United Kingdom, disease modelling

## Abstract

A combined *Haemophilus influenzae* type b (Hib)/meningococcal serogroup C (MenC) vaccine will soon be unavailable in the UK immunisation schedule due to discontinuation by the manufacturer. An interim statement by the Joint Committee on Vaccination and Immunisation (JCVI) advises stopping MenC immunisation at 12 months of age when this occurs. We undertook an analysis of the public health impact of various potential meningococcal vaccination strategies in the UK in the absence of the Hib/MenC vaccine. A static population-cohort model was developed evaluating the burden of IMD (using 2005–2015 epidemiological data) and related health outcomes (e.g., cases, cases with long-term sequelae, deaths), which allows for the comparison of any two meningococcal immunisation strategies. We compared potential strategies that included different combinations of infant and/or toddler MenACWY immunisations with the anticipated future situation in which a 12-month MenC vaccine is not used, but the MenACWY vaccine is routinely given in adolescents. The most effective strategy is combining MenACWY immunisation at 2, 4, and 12 months of age with the incumbent adolescent MenACWY immunisation programme, resulting in the prevention of an additional 269 IMD cases and 13 fatalities over the modelling period; of these cases, 87 would be associated with long-term sequelae. Among the different vaccination strategies, it was observed that those with multiple doses and earlier doses provided the greatest protection. Our study provides evidence suggesting that the removal of the MenC toddler immunisation from the UK schedule would potentially increase the risk of unnecessary IMD cases and have a detrimental public health impact if not replaced by an alternate infant and/or toddler programme. This analysis supports that infant and toddler MenACWY immunisation can provide maximal protection while complementing both infant/toddler MenB and adolescent MenACWY immunisation programmes in the UK.

## 1. Introduction

Invasive meningococcal disease (IMD) is caused by *Neisseria meningitidis* and historically has five predominant disease-causing serogroups (A, B, C, W, and Y), each with a unique epidemiological profile [1]. In general, IMD is unpredictable, and its incidence rates can vary across age groups, geographic regions, and over time [2]. Although IMD is relatively uncommon, the consequences can be severe, leading to serious long-term sequelae or death [3]. In England, the estimated overall IMD case fatality rate in the epidemiological year 2019/2020 was 7% [4]. In addition to the substantial public health consequences, the economic burden of IMD is considerable, as cases lead to high health resource utilisation and productivity losses among patients and caregivers [5]. Due to these aspects, protection across the entire population against IMD is a key public health consideration.

In 1999, the UK was the first country to introduce serogroup C (MenC) conjugate vaccines into an infant national immunisation programme (NIP), along with a comprehensive catch-up programme for those up to 18 years of age [6]. The MenC NIP was optimised at multiple time points from the initial infant schedule alone and through various infant prime and toddler (12 months of age) booster schedules. In 2013, an adolescent MenC dose at 13/14 years of age was included in the NIP with the aim of providing indirect (herd) protection across the entire population, in addition to providing direct protection for vaccinated adolescents [6]. In 2015, the adolescent MenC NIP was switched to a serogroups A, C, W, and Y (MenACWY) conjugate vaccine, driven by increases in serogroup W (MenW) cases [6]. In the same year, the UK became the first country to implement a serogroup B (MenB) vaccine into the infant NIP along with a booster dose at 12 months of age. This then led to the removal of the last remaining infant MenC dose from the NIP, leaving MenC immunisation at 12 months of age and MenACWY immunisation in adolescents [7].

All of the UK meningococcal NIPs continue to achieve high vaccine uptake rates, as exemplified by the most recent data (from epidemiological year 2020–2021) reporting that 92% of English infants receive two priming doses of the MenB vaccine [8]. Similarly, 87% of English adolescents received the MenACWY vaccine as part of the NIP, demonstrating the success of the school-based immunisation programme [8]. These high vaccine uptake rates coupled with high vaccine effectiveness have delivered a significant public health impact by reducing IMD cases [9]. The introduction of the MenC NIP resulted in an approximate 96% decline in MenC cases in England from almost 1000 cases in the epidemiological year prior to introduction to around 30–40 cases per year following implementation [8]. The MenB NIP reduced MenB cases by 75% in vaccine-eligible English children [10], and the MenACWY vaccine had a 94% vaccine effectiveness against MenCWY IMD in English adolescents [6]. Furthermore, the MenACWY adolescent NIP was shown to provide indirect protection across other age groups that were not offered vaccine [6,11]. Despite these achievements, clusters and outbreaks of IMD are periodically observed in the UK, reinforcing the need for robust public health and prevention strategies [12,13].

The current UK meningococcal vaccine schedule consists of the MenB vaccine at 2, 4, and 12 months of age; *Haemophilus influenzae* type b (Hib)/MenC conjugate vaccine (Hib/MenC) at 12 months of age; and MenACWY vaccine at approximately 14 years of age [14]. However, changes to this schedule are required due to the discontinuation of the Hib/MenC conjugate vaccine (Menitorix^®^), with supply expected to be exhausted in 2024/2025, and because there is no equivalent vaccine available. The Joint Committee on Vaccination and Immunisation (JCVI) recently published an interim statement advising that once the Hib/MenC vaccine is withdrawn from the schedule, a replacement Hib-containing vaccine should be introduced at either 12 or 18 months of age, but that there was to be no replacement for the MenC immunisation. The rationale for this decision is that infants and toddlers can rely on indirect protection afforded by the MenACWY adolescent programme [15]. However, reliance on indirect protection leaves the age groups traditionally with the highest IMD incidence without direct protection. This potentially leaves them susceptible to disease and can explain why sporadic cases and clusters of MenC, MenW, and MenY disease have occurred in these age groups whilst adolescent immunisation programmes have been in place [16].

Evidence-driven advocacy groups such as the Meningitis Research Foundation have expressed concern about the removal of the MenC vaccine from the toddler schedule with regard to the impact on IMD cases, and have been seeking clarification for the evidence base underlying this recommendation [17]. Other options that JCVI had previously discussed included providing extra protection against additional serogroups through the MenACWY immunisation of infants and/or toddlers. A particular benefit of these options highlighted in JCVI discussions was the opportunity to increase MenW protection and minimise the impact of future MenW outbreaks [11,17,18]. Modelling the public health impact of various IMD vaccination strategies in the absence of the Hib/MenC vaccine would therefore be beneficial to inform the optimisation of the UK immunisation schedule.

Meningococcal immunisation schedules have continued to evolve as new vaccines become available, and there is an improved understanding of indirect protection, cross-protection, antibody persistence, and different dosing schedules. The optimal meningococcal immunisation schedule is still to be determined, likely to be country or region specific due to underlying epidemiology and will likely require future adaption and evolution. In this context, the public health modelling of different IMD vaccination strategies becomes ever more important. Since the discontinuation of the Hib/MenC vaccine was first reported in February 2020 [19], there has been an interest in understanding and modelling the impact of meningococcal vaccine schedules in the absence of this vaccine. In this context, we set out to analyse the public health impact of various paediatric meningococcal immunisation strategies in the UK.

## 2. Methods

### 2.1. Model Structure

To inform the model structure for evaluating the burden of IMD and related health outcomes, a review of best modelling practices for meningococcal disease and vaccination models was undertaken [20,21]. Of the 21 IMD models reviewed, 13 were static models that had previously been found to be more conservative than a dynamic modelling approach [22]. For the purposes of this analysis, and to not bias the results in favour of vaccination, a conservative modelling approach was considered most appropriate and, therefore, a static model was chosen. The static multi-cohort population model was developed in Microsoft Excel^®^ to evaluate the public health impact of different immunisation strategies while simulating the clinical course of IMD in the full UK population.

The model estimates the size of the population each year and at every age, based on the number of inhabitants in the UK. In every age group, the numbers of serogroup A, C, W, and Y cases were calculated. Cases are reduced by the effectiveness of the vaccination strategy in the model. The model structure is presented in Figure S1.

The model considers the health implications of IMD cases with acute disease, and the long-term complications that occur after the acute disease phase. Thus, in addition to evaluating the quality-adjusted life-year (QALY) decrements occurring during the acute disease phase, the health decrements occurring due to the long-term effects of IMD or developed sequelae are evaluated. The consequences of death related to IMD, either during the acute disease phase or after the acute disease phase, were also considered.

### 2.2. Vaccination Strategies

The model allows for a comparison of any two meningococcal immunisation strategies, which can include multiple vaccinations in the following three different age groups: infants, toddlers, and adolescents. MenACWY immunisation strategies providing direct protection against the respective serogroups contained in the vaccine are modelled. In each immunisation strategy, multiple doses can be modelled, and the indirect effect from adolescent MenACWY immunisation is incorporated into estimates of vaccine effectiveness.

Potential UK immunisation strategies were designed to explore those which could supersede the current 12 months of age MenC toddler dose, including the recent JCVI recommendation to stop the MenC immunisation of toddlers and rely on indirect protection from the adolescent MenACWY programme; the additional strategies were selected based on initial options raised by JCVI, the consideration of existing infant and toddler immunisation schedules for other infectious diseases, and the logistical feasibility of the immunisation schedule. For example, the JCVI noted that infant MenACWY immunisation may provide important direct protection against MenW, that infants might require two doses of the MenACWY vaccine for sufficient protection, and that a single MenACWY dose in infancy could be better than a toddler MenACWY dose [18]. The immunisation strategies and vaccine uptake assumptions modelled in this analysis are listed in Table S1. The vaccine uptake inputs by age represent compliance with the vaccine schedule, i.e., number of doses in a series, rather than uptake at a particular age. The model conservatively assumed that compliance decreased with each dose. The more doses included in a schedule, the lower the uptake compared with the uptake in a one-dose schedule. Therefore, in strategy 4, which involved one dose at 12 months of age, a 96% uptake was assumed, while in strategy 5, which involved two doses (at 5 and 12 months of age), the uptake of the 12-month dose was 89% to reflect the expected decrease in compliance. For the purposes of this analysis, all immunisation strategies are compared to a future scenario without infant or toddler MenC or MenACWY immunisation but with an adolescent MenACWY vaccine programme.

### 2.3. Vaccine Effectiveness

Vaccine effectiveness depends on the number of doses administered and the age of the vaccine recipients. The effectiveness of the infant MenACWY vaccine is assumed to be 85% and 92% for the first dose and second doses, respectively. The 12-month doses of the MenACWY vaccine are assumed to have 92% effectiveness [23], and adolescent MenACWY immunisation is assumed to have 94% effectiveness [6]. The waning of vaccine effectiveness is also included in the model; full protection is initially assumed for a defined period, and, after this period, the effectiveness exponentially wanes [24,25]. The model also incorporates a static approximation of the indirect protection of MenACWY immunisation as adolescent immunisation is included in all modelled schedules. The model assumes that the indirect effect reduces the number of IMD cases in the unvaccinated population by 50% due to the indirect effect the adolescent vaccination programme provides [11]. The inclusion of indirect protection is aligned with current JCVI expectations that infants and toddlers will receive some indirect protection against MenC (as well as MenAWY) [15]. Cross-protection from MenB immunisation to MenACWY IMD is not incorporated.

### 2.4. Population

The model considers the full UK population, including the population born during the model time horizon and the population alive at the beginning of the model time horizon. Both populations are followed lifelong, i.e., until the age of 100 years. The model assumes that the population is stable such that the number of newly born children each year is the same. The size of the population age groups that are alive at the beginning of the model time horizon is based on real-world UK data.

### 2.5. Time Horizon

The time horizon of the model is 30 years. Therefore, new population cohorts enter the model for 30 years, representing the duration of the immunisation programme. These cohorts are followed in the model until death, which is assumed to occur at no older than 100 years of age.

### 2.6. Model Perspective

The model includes both a decision-maker perspective, which considers the direct effects of IMD, and a societal perspective, which captures both direct and indirect consequences of IMD.

### 2.7. Societal Effects: Patients and Caregivers

The societal effects of IMD included in the model are categorised as either effects on patients or effects on caregivers. Patient effects included in the model are the QALY loss experienced by a patient after acute IMD, whether long-term sequelae develop, and QALY loss of caregivers at different stages (during the acute phase of IMD, if patients develop long-term sequelae or if patients die from IMD). The model also allows each patient to have more than one caregiver; the base case assumes that each patient has 2 caregivers [26]. Caregivers are assumed to be 44 years old based on the sum of both the average age at childbirth, which is 31 years, and the average age of IMD diagnosis in the UK, which is 13 years [27].

### 2.8. Discounting

Discounting was applied to health outcomes, as the model accumulates health outcomes for 100 years. The discount rate applied to health outcomes in the base case is 3.5%, with sensitivity analyses recommending a 1.5% discount rate for each based on the National Institute for Health and Care Excellence (NICE) guidelines [28].

### 2.9. Model Outcomes

The model evaluates the health outcomes associated with IMD. These are the number of IMD cases, manifestation, and number or deaths, each stratified by serogroup. The model also calculates the QALY, and life-year (LY) losses associated with IMD. The QALY loss outcomes are separated into the QALY losses of the patients and the QALY losses of the caregivers, as well as the reason for the QALY loss (i.e., QALY losses of patients and caregivers during the acute phase, long-term QALY losses of patients without sequelae, long-term QALY losses of patients and caregivers if patients developed sequelae, QALY losses of patients if patients died of IMD, QALY losses of caregivers if patients died of IMD, and QALY losses of patients due to early death after IMD).

## 3. Model Inputs

### 3.1. Targeted Literature Reviews

Targeted literature reviews were conducted to collect the latest available evidence concerning the burden of IMD, immunisation programmes, health resource utilisation, and societal effects to identify values for the model parameters. Where possible, UK-specific inputs were used in the model; when UK-specific data were not available, robust inputs were obtained from other geographical regions that could be expected to be generalisable to the UK.

### 3.2. Epidemiological Data

The model uses epidemiological data to calculate and categorise the number of IMD cases in the UK. The number of UK inhabitants separated into 10 age groups (0–11 months, 12–23 months, 2–4 years, 5–9 years, 10–14 years, 15–22 years, 23–24 years, 25–44 years, 45–64 years, and 65–100 years) was included in the model based on the 2021 population data reported by the Office for National Statistics [29]. The population size in each age group is reported in Table S2.

IMD incidence data differ across the serogroups and age groups. To provide a stable period for the estimation of MenACWY IMD incidence prior to the introduction of the MenACWY vaccine, the average incidence of each serogroup from 2005 to 2015 was used to inform the base case (ECDC). Using incidence data collected after 2015 could result in double counting the direct and indirect effects of the MenACWY adolescent routine and catch-up immunisation programmes. The early portion of this 10-year period was prior to the increases in MenW; therefore, the impact of increasing MenW cases at the very end of the period is mitigated. Additionally, MenC indirect protection was incumbent across the 10-year period, meaning that the MenC case numbers modelled are conservative and likely lower than would have been observed without a herd effect. Briefly, across all age groups, there were no cases of IMD caused by serogroup A. The 0–12 months age group displayed the greatest IMD incidence caused by MenC (0.15/100,000 population), MenW (1.12/100,000 population), and MenY (0.62/100,000 population). The IMD incidence in the other age groups was in the ranges of 0.04–0.07/100,000 population (MenC), 0.02–0.24/100,000 population (MenW), and 0.03–0.26 (MenY) (Table S2). During this 10-year period, MenC case numbers were likely controlled by the incumbent MenC immunisation programme in infants and toddlers, by the indirect protection induced by the original MenC catch-up campaign, and lastly, from 2013, by the implementation of adolescent MenC immunisation. This routine MenC adolescent programme was subsequently switched to the MenACWY vaccine in late 2015 along with the commencement of a MenACWY vaccine catch-up programme in those 14–18 years of age. MenA cases have not been detected in the UK for many years, but given the unpredictability of IMD and that MenA still occurs in some parts of the globe, the protection offered by the MenACWY vaccine remains important [4,30]. IMD is an unpredictable disease with a large variation in incidence, and examination revealed peaks that were used to inform high-incidence scenarios of MenC (average of 2017–2018) and MenW (average of 2016–2018). No clear peaks in MenY incidence were observed; therefore, a hypothetical MenY peak, proportional to MenW, was created.

### 3.3. Clinical Inputs

The clinical inputs in the model consist of vaccine effectiveness (consisting of vaccine effectiveness, waning of vaccine effectiveness, and indirect protection inputs), mortality, and sequelae. The distribution of IMD cases by clinical manifestation and serogroup were also considered, and the input values of these parameters are shown in Tables S3–S5. Age-related mortality during the acute phase of IMD was captured by the case fatality rates and included in the model. Briefly, the greatest case fatality probability (22.8%) occurred in the 65–100 years age group, followed by the 45–65 years age group (8.5%), 25–45 years age group (7.1%), and 23–25 years and 15–23 years age groups (6.8% each). The case fatality probability in the younger age groups ranged from 4.7% to 4.9% (Table S5). The probability of long-term sequelae, including amputation, anxiety, arthritis, cognitive impairment, depression, hearing loss, migraine, motor deficits, neurological disability, renal failure, seizure, skin scarring, speech problems, and visual impairment, were included in the model, as presented in Table S6.

### 3.4. Disutility Inputs

IMD patients and their caregivers may also experience a decrease in their quality of life. During the acute disease phase, regardless of the serogroup or manifestation, patients’ utilities decrease by 0.2, while the caregivers’ utilities do not change [31]. IMD may also lead to a reduction in quality of life after the acute disease phase. In the model, utility decrements of patients after the acute IMD phase are considered. The size of the decrement depends on whether the patients develop sequelae. Additionally, caregivers may experience a lower quality of life after acute IMD if patients develop long-term severe sequelae. Furthermore, if the patient dies of IMD, the bereaved caregivers also experience disutility. These utility decrements after acute IMD used in the model are presented in Table S7. In addition to the disutilities associated with long-term sequelae, the JCVI also recommended that a quality adjustment factor (QAF) can be applied to patient QALY gains due to averting long-term sequelae, and a QAF multiplier of three is incorporated into the base case analysis [9,20]. The rationale for including the QAF in IMD modelling is that health utility losses vary widely among individuals because of the diversity in the number and severity of IMD-associated sequelae, and it is plausible that health benefits are underestimated in IMD models [20].

## 4. Analysis Approach

The base case analysis was conducted from a UK societal perspective using the settings and inputs previously described. All potential immunisation strategies were compared with an adolescent alone MenACWY programme (without any infant or toddler MenC or MenACWY immunisation), which reflects the most recent JCVI advice in response to the withdrawal of the Hib/MenC vaccine [15]. Additional deterministic sensitivity analyses and scenario analyses were conducted to explore the validity and robustness of the results and the key drivers of the clinical impact of various MenACWY immunisation strategies (see Table 1).

## 5. Results

### 5.1. Public Health Impact

**Figure 1:**
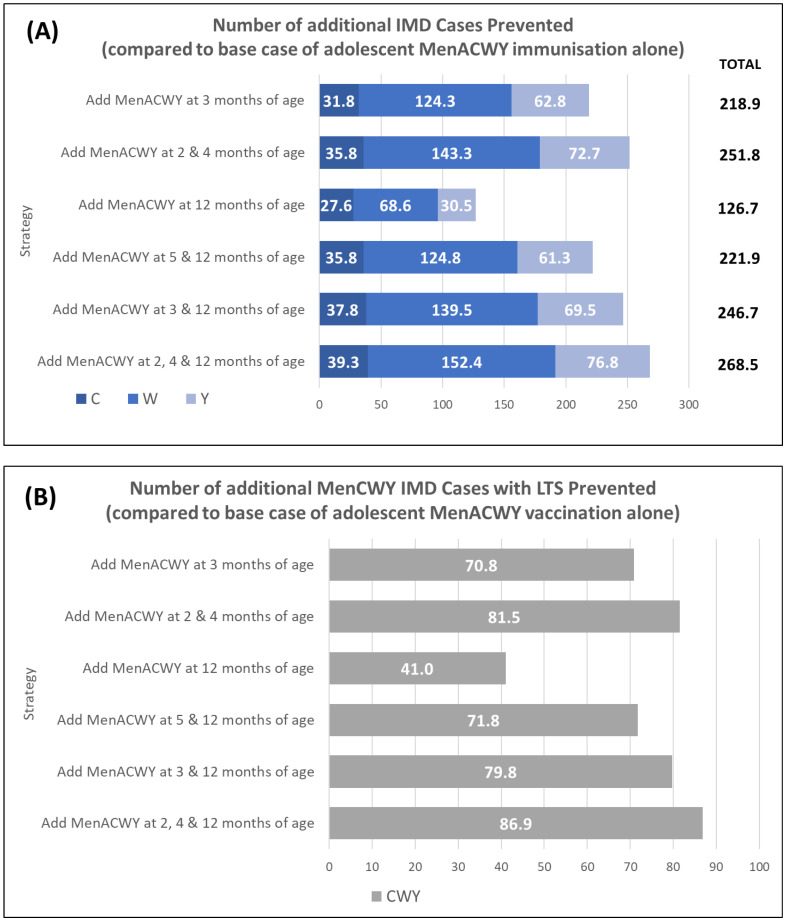

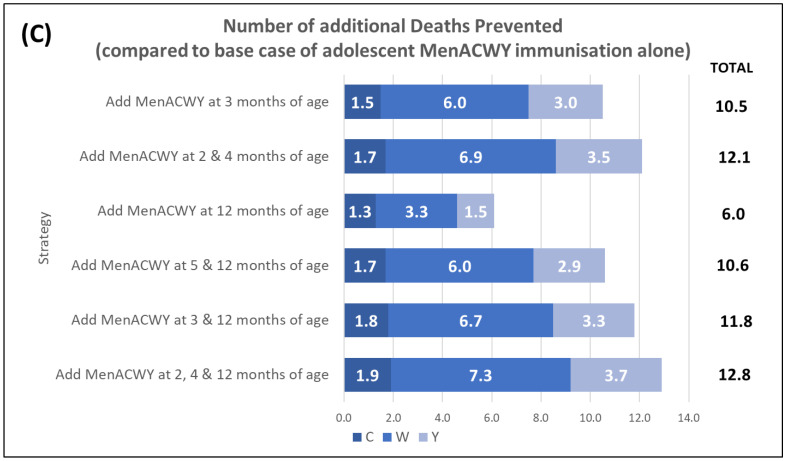
Public health impact of various MenACWY immunisation strategies on IMD cases compared with base case of adolescent MenACWY immunisation alone. Impact was assessed as (**A**) number of additional IMD cases prevented (versus the base case); (**B**) number of additional cases with long-term sequelae (LTS) prevented; and (**C**) number of additional deaths prevented. Note that values for individual serogroups may not equate to the total value due to rounding.

The modelling of each of the six immunisation strategies compared with only adolescent MenACWY immunisation demonstrates that each of the strategies provides an incremental benefit with respect to preventing cases, long-term sequelae, and deaths over the model time horizon (see Figure 1). The greatest benefit is observed when the MenACWY vaccine is also given at 2, 4, and 12 months of age, with 269 additional IMD cases and 13 IMD-related deaths prevented; of these cases, 87 are associated with long-term sequelae. Even the least impactful of the six strategies explored (MenACWY vaccine at 12 months of age) resulted in an additional 127 IMD cases and 6 IMD-related deaths prevented compared with the adolescent MenACWY immunisation programme alone.

### 5.2. High-Incidence Scenarios

Different high-incidence scenarios of MenC, MenW, and MenY IMD were explored to understand the public health impact of the different immunisation strategies in hypothetical situations in which incidence rates peak (Table 2). The immunisation strategy offering the greatest impact is the combination of the MenACWY vaccine at 2, 4, and 12 months of age with the adolescent programme. This would prevent approximately 774 additional IMD cases and 37 IMD-related deaths, including 250 cases of long-term sequelae over the modelling period in the case of high MenCWY incidence compared with adolescent immunisation alone.

### 5.3. Deterministic Sensitivity Analyses

The parameters considered most important in the UK context and the parameters with the greatest uncertainty were evaluated in deterministic sensitivity analyses comparing MenACWY immunisation at 3 and 12 months of age, in addition to adolescent MenACWY vaccination alone (see Table 1). The most influential variables were removing the QAF from the analysis, decreasing the discounting rate, changing the model perspective, and removing broader caregiver elements. The sensitivity analysis using global values, obtained from an Italian study [32], demonstrates the importance of using UK-specific data when possible, and these values were used in the base case analysis [9].

### 5.4. Scenario Analyses

**Figure 2:**
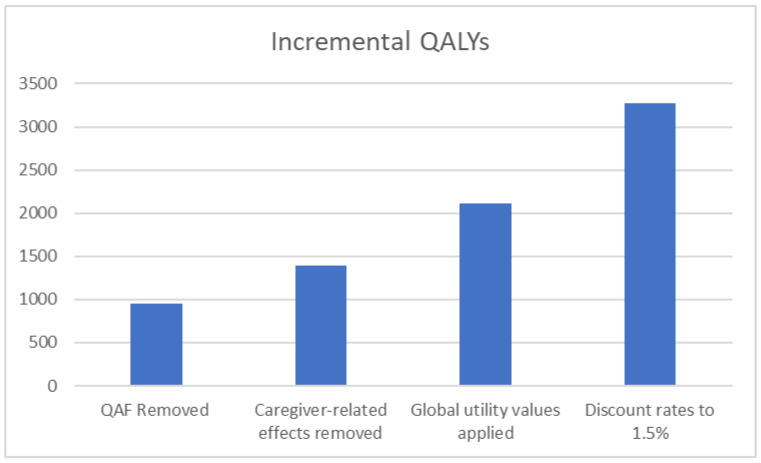
Sequential stepwise analysis showing the impact of MenACWY immunisation in adolescents and at 3 and 12 months of age compared with adolescent MenACWY immunisation alone on incremental QALYs as the model settings and parameters change.

A subsequent sequential scenario analysis was also conducted to explore the public health impact of MenACWY immunisation at 3 and 12 months of age in addition to adolescent MenACWY immunisation alone by applying the most influential and robust modelling assumptions identified in prior deterministic sensitivity analyses to additionally understand the most optimistic impacts of such an immunisation programme. When considering the addition of the MenACWY vaccine at 3 and 12 months of age, the resulting impact on QALYs after cumulatively considering a change in the modelling perspective, peak incidence rates (MenC, MenW, and MenY), application of the QAF (as recommended by the JCVI), and UK discounting rates recommended by NICE for use in sensitivity analyses (1.5% for benefits) is shown in Figure 2.

## 6. Discussion

### 6.1. Public Health and Policy Implications of Stopping 12-Month MenC Immunisation

The recent JCVI recommendation to halt toddler 12-month MenC immunisation [15] comes at a time when there is considerable uncertainty and unpredictability surrounding how IMD incidence will evolve following the COVID-19 pandemic. IMD remains associated with significant morbidity and mortality, which can be reduced through further optimisation of meningococcal immunisation efforts [3,33]. In this modelling study, we identified that using the MenACWY vaccine in UK infants and/or toddlers and assuming no change in MenACWY epidemiology would potentially prevent up to an additional 269 IMD cases compared with the adolescent MenACWY immunisation programme alone following the removal of the 12-month MenC vaccine dose. An even greater benefit could potentially be achieved if the meningococcal epidemiology changed, and there were increases in the MenC, MenW, or MenY incidence, as have been observed in the past. Previous modelling studies assessing the impact of MenACWY vaccination also included vaccination with 4CMenB [34,35]. Therefore, it is difficult to compare our results to the results of these studies. Nevertheless, our study is consistent with a previous study conducted in Canada showing that vaccination at 12 months with MenACWY followed by a booster dose at adolescence was predicted to result in a 70% reduction in IMD cases over a 40-year horizon, representing the most effective strategy [36]. While the previous analysis was restricted to vaccination at 12 months and in older age groups, our model considered vaccination in infancy, further contributing to the published literature.

The importance of reducing the burden of IMD is recognised not only in the UK but also globally. Through a collaborative, multi-disciplinary process, the WHO created a global roadmap for eliminating bacterial meningitis epidemics and reducing deaths and disability associated with bacterial meningitis, including IMD, by 2030. A key objective of this roadmap is reducing bacterial meningitis cases and deaths by 50% and 70%, respectively [30]. These targets apply to IMD and are relevant for all countries, such as the UK, and are not just restricted to countries traditionally associated with high incidence rates and epidemics, such as those in Sub-Saharan Africa. Meningococcal immunisation is identified in the roadmap as a key strategic priority for achieving these reductions in IMD cases and deaths [2,30]. However, the implementation of immunisation programmes is often influenced by many factors, including vaccine availability, IMD incidence, and the severity of disease [16].

In addition to the usual unpredictability associated with IMD, the COVID-19 pandemic introduced additional uncertainty to meningococcal epidemiology [37,38]. School closures due to COVID-19 impacted the school-based immunisation programme in the 2019/2020 academic year, resulting in reduced vaccination rates [39]. With the lower immunisation rates due to COVID-19–related lockdowns and social distancing measures, it is unclear how the IMD incidence rates will rebound and evolve in the coming years [40]. IMD cases were few during the COVID-19 pandemic, which was initially presumed to be due to reduced transmission and carriage as result of social distancing measures. Based on this assumption and coupled with the reduced carriage rates due to adolescent MenACWY immunisation, IMD incidence in the UK was expected to remain low for several years with vaccines possibly being deprioritised [41]. However, new evidence has showed that meningococcal carriage was not necessarily impacted by COVID-19–related social distancing [42], and a rise in UK adolescent/adult MenB cases has been observed much more quickly than expected. For example, Clark et al. [43] reported that in England during the period following the relaxation of COVID-19 lockdown measures, MenB IMD cases in adolescents and young adults not only returned to pre-pandemic levels but rapidly exceeded them. An alternative hypothesis that may contribute to the explanation of low IMD incidence during the COVID-19 pandemic includes the suppression of seasonal respiratory viruses, such as influenza, which are associated with increased carriage and IMD [37,38,44]. Similar increases in childhood invasive pneumococcal disease (IPD) have been observed in the UK following the COVID-19 pandemic, despite modelling predictions that there would be a long-term reduced incidence [45]. This rapid re-emergence of IPD in UK children now exceeds pre-pandemic levels, and has also coincided with the return of seasonal respiratory viruses [46]. Data from Israel and Belgium now also suggest that the COVID-19 pandemic did not impact pneumococcal carriage [45,47,48,49]. Therefore, given the unpredictability of IMD incidence, the fact that no cases of IMD were observed in the epidemiological year 2020/2021 is not necessarily predictive of the IMD incidence in future years because this period is within the pandemic period, and IMD incidence during the pandemic is unlikely to reflect the post-pandemic incidence. As the evidence base of IMD cases and patterns of other infectious diseases (e.g., respiratory syncytial virus, IPD) emerge, greater accuracy can be incorporated in immunisation modelling approaches. In the interim, assumptions that IMD incidence will remain low for many years because of the COVID-19 pandemic should potentially be viewed with caution. Nevertheless, COVID-19-related changes in vaccination coverage and IMD incidence represent a perspective that should be considered in the decision-making process along with IMD unpredictability.

The current UK meningococcal immunisation programmes have had a significant public health impact [6,10]. However, supplementary approaches to reducing IMD, minimising the impact of outbreaks, and more fully protecting the population must be considered. This is especially true in a resource-constrained healthcare setting, where approaches that reduce stress and strain on the healthcare system are valued. The future UK schedule where direct protection against MenACWY IMD is provided only to adolescents will result in all other age groups relying solely on indirect protection. This means that the younger and most vulnerable age groups for MenACWY disease are not sufficiently protected, which results in an increased risk of disease compared with the current schedule, in which the MenC vaccine is offered to toddlers. Case fatality rates of approximately 5% in individuals under 15 years [33] highlight that serious morbidity and mortality can be further averted by providing direct protection through an early childhood MenACWY immunisation programme. 

All of the infant-alone, toddler-alone, and infant and toddler–combined MenACWY immunisation schedules modelled in this study prevented additional IMD cases compared with the adolescent-alone MenACWY immunisation programme. Including a single dose of the MenACWY vaccine at 12 months of age could prevent approximately 127 additional MenCWY cases compared with an adolescent-alone MenACWY immunisation programme. This would equate to approximately 12 fatalities and 71 cases of long-term sequalae prevented. Due to the higher IMD incidence in infants, it was not surprising that all schedules incorporating infant MenACWY immunisation prevented greater numbers of IMD cases than those achieved with only toddler and adolescent immunisation schedules. This ranged from a 0.73-fold increase in the number of cases prevented by using the MenACWY vaccine at 3 months of age (and adolescence) to a 1.12-fold increase in cases prevented by a 2-, 4-, and 12-month schedule (and adolescence). The most impactful strategy of implementing MenACWY at 2, 4, and 12 months of age would result in approximately 269 fewer IMD cases, which would be associated with 13 fewer fatalities and 87 cases of long-term sequalae when compared with an adolescent-alone MenACWY immunisation programme. These analyses are conservative and likely underestimate the number of IMD cases prevented, since the base case considers MenC incidence estimates gained from a period where MenC IMD was well controlled. This was via the incumbent MenC infant and toddler immunisation programme and robust indirect protection afforded by the original large-scale catch-up programme. The modelling of high incidence MenC, MenW, MenY, and MenCWY IMD scenarios further demonstrated the benefit of all the infant and/or toddler MenACWY immunisation schedules investigated. These findings are relevant due to the unpredictable nature of meningococcal epidemiology and the fact that these high-incidence scenarios were based upon previous peaks of disease experienced in the UK.

Although tempered by the indirect protection afforded by the adolescent MenACWY immunisation, the removal of the UK toddler MenC immunisation programme with no replacement will potentially have a detrimental impact on public health in the UK [15,27]. Not only will direct MenC protection in toddlers be lost, but an opportunity to implement the MenACWY vaccine in infants and/or toddlers providing direct protection will be missed. This is especially a concern at a time when social interactions have started to increase again as COVID-19 lockdown–related restrictions have eased and infectious disease transmission has increased, creating a potentially vulnerable population [8,43,48,50]. It is also important to contrast the withdrawal of the MenC vaccine from the NIP to the WHO roadmap goals, requiring additional meningococcal vaccine use to reduce IMD cases in all countries, including the UK. Questions remain as the JCVI advice to cease toddler MenC immunisation [15] does not indicate how concerns from earlier discussions with respect to MenW-related deaths and the requirement for direct protection via infant MenACWY vaccination have been resolved [18].

The JCVI decision is based on the belief that indirect protection from the adolescent MenACWY immunisation programme will be sufficient to control MenACWY cases and deaths across the UK population. However, our analysis clearly shows the potential benefit of the direct protection of infant and/or toddler MenACWY immunisation in addition to indirect protection, or possible cross-protective effects from MenB vaccines [11,42,51]. These findings were generated from a conservative modelling approach incorporating an indirect effect of 50%, as supported by Carr 2022 [11], and still show that, even in this scenario, deaths and cases would be averted with an infant and/or toddler MenACWY immunisation programme.

### 6.2. Study Limitations

Several limitations are identified in this study; however, they are typically factors that underestimate the full value of meningococcal immunisation programmes, as we maintained a conservative approach in our analyses. Creating statistical models that accurately represent infectious diseases can be challenging, as all mathematical models are essentially a simplification of real-world conditions [20,21] and heavily rely on the availability of robust data to populate the model. For example, the assumptions used to calculate the indirect effect were simplified for incorporation into this static multi-cohort population model; however, the simplifying assumptions were usually conservative [22]. Additionally, due to the uncommon nature of IMD, UK-specific inputs were not always available with the degree of granularity permitted in the model (e.g., breakdown of case fatality rates by serogroup or utility decrements by manifestation). Approaches to estimating and incorporating more innovative broader value elements, such as caregiver QALY losses due to IMD deaths or long-term sequelae, are included in the model but have not been fully aligned on and robustly reported in the literature [9,52]. Furthermore, projecting the impact of such changes over a long-time horizon encompasses substantial uncertainty for an unpredictable disease, such as IMD.

Not all of the immunisation schedules modelled in our study are currently licensed, such as the single-dose priming schedules in infants aged less than 6 months. However, there are studies ongoing that investigate some of these, and the JCVI has previously discussed these as options to be considered. The modelled strategies do not consider a potential cross-protection of the infant and toddler MenB vaccine used in the UK as there is an ongoing debate on the level and duration of cross-protection, which depends on the proteins expressed by MenACWY strains responsible for causing disease in the UK. The cross-protection of MenACWY strains can potentially be considered as an additional benefit in the absence of meningococcal conjugate vaccines, but should not be a substitute for the anti-capsular protection offered by conjugate vaccines. This is emphasised by the conflicting data showing the potential of the MenB vaccine to provide protection against MenW strains [51], contradictory to JCVI discussions highlighting the need for direct protection from MenACWY vaccines in infants to prevent the MenW cases that are occurring [18]. The cases of MenCWY IMD, which continue to occur in UK infants and toddlers after the introduction of the MenB vaccine, also highlight why this cross-protection was not considered in the model and indicate the need for direct protection from MenACWY vaccines [4,8].

The focus of this study was the potential health impact of replacing the Hib/MenC vaccine (due to its discontinuation) with different combinations of infant and/or toddler MenACWY immunisation schedules in the UK. Here, the outcomes of interest included the numbers of IMD cases, cases with long-term sequelae, and deaths. This approach enabled us to determine that the removal of MenC toddler immunisation from the UK schedule would increase the risk of unnecessary IMD cases and have a detrimental public health impact if not replaced by an alternate infant and/or toddler programme. Furthermore, we determined that the most effective strategy would be immunisation with MenACWY at 2, 4, and 12 months of age based on the outcomes of interest. However, we did not conduct a cost-effectiveness analysis of the different immunisation programmes. Such analyses could be of interest to stakeholders and decision makers, and represent a topic that could be addressed in future studies.

Additionally, the potential indirect impact (herd effect) of infant and/or toddler MenACWY immunisation on the elderly, representing another vulnerable population [33], was not explored in this study. Meningococcal carriage in infants and toddlers is considered rare as these younger age groups are not important transmitters. Therefore, vaccinating these younger age groups is not considered to indirectly protect older age groups. In contrast, adolescents have been shown to have the highest meningococcal transmission and carriage rates [53]. Adolescent vaccination with MenACWY has been shown to confer herd protection in the UK [11], and has the greatest potential to control IMD in other age groups, such as the elderly [53]. Nevertheless, indirect protection among the elderly is an important concern, representing another topic that could be explored in future studies.

### 6.3. Study Strengths

The key strength of the present study is that the analysis comprehensively considers the impact of the recent JCVI decision (i.e., remove the Hib/MenC vaccine at 12 months and not replace the MenC component versus inclusion of an MenACWY vaccine in infancy into the NIP) on IMD in the UK. This is the first published analysis to consider different possible immunisation scenarios and their public health impact in the absence of the Hib/MenC vaccine in the UK. Additionally, the modelling approach allows multiple perspectives to be considered while incorporating standard public health outcomes such as IMD cases, conventionally accepted societal elements such as patient disutilities, and broader value elements such as parental or caregiver QALY losses associated with the IMD-related death of a child. These are all aligned with newer and more innovative approaches to modelling [54].

The results of these analyses can be used to inform and support not only UK-based immunisation policies, but also worldwide efforts led by the WHO to evaluate meningococcal vaccination strategies with the aim of informing evidence-based policies and optimising IMD prevention.

## 7. Conclusions

The recent announcement of the withdrawal of the Hib/MenC vaccine from the UK market has sparked some discussion. As a result, the JCVI has decided not to replace the MenC component for the direct protection of toddlers against MenC but to rely on the indirect protection offered by the adolescent MenACWY program.

Our study provides evidence suggesting that the removal of the MenC vaccine from the UK toddler immunisation schedule would potentially increase the risk of unnecessary IMD cases, many of which are likely in infants/toddlers. A combined infant and toddler MenACWY immunisation programme would potentially have an even greater public health impact in the UK while complementing both the established infant/toddler MenB and adolescent MenACWY immunisation programmes. Immunisation with MenACWY at 2, 4, and 12 months of age is the strategy that would likely be associated with the greatest public health impact if implemented, as evidenced by a reduction in IMD cases and deaths.

## Figures and Tables

**Table 1:** Impact of various deterministic sensitivity analyses on incremental QALYs when comparing MenACWY immunisation in adolescence and at 3 and 12 months of age versus base case of adolescent MenACWY immunisation alone.

Sensitivity Analysis	Results (Incremental QALYs)	Impact on QALYs vs. Base Case
**Base Case** (adolescent MenACWY immunisation alone)	**1618**	**---**
Quality adjustment factor (QAF) removed from the analysis	946	Unfavourable
Removed caregiver-related effects	1387	Unfavourable
Global utility values applied	2113	Favourable
Changed discount rates to 1.5% benefits	3268	Favourable

**Table 2:** Public health impact and number of MenCWY IMD cases prevented under different high-incidence scenarios of various MenACWY immunisation strategies compared with base case of adolescent MenACWY immunisation alone.

Strategy	Standard Incidence of MenCWY (Reference Point)	High MenC Incidence	High MenW Incidence	High MenY Incidence	High MenCWY Incidence
Additional IMD Cases Prevented (compared with base case of adolescent MenACWY immunisation alone)					
Add MenACWY at 3 months of age	218.9	316.2	427.2	325	630.7
Add MenACWY at 2 and 4 months of age	251.8	365.8	489.6	373.4	725.2
Add MenACWY at 12 months of age	126.7	159.7	268.3	193.5	368.1
Add MenACWY at 5 and 12 months of age	221.9	311.5	441.3	331.6	640.5
Add MenACWY at 3 and 12 months of age	246.7	351.5	486	367.5	711.6
Add MenACWY at 2, 4, and 12 months of age	268.5	387.1	524.8	389.8	773.8
Additional Long-Term Sequelae Cases Averted (compared with base case of adolescent MenACWY immunisation alone)					
Add MenACWY at 3 months of age	70.9	102.3	138.2	105.2	204.1
Add MenACWY at 2 and 4 months of age	81.5	118.3	158.4	120.8	234.6
Add MenACWY at 12 months of age	41	51.7	86.8	62.6	119.1
Add MenACWY at 5 and 12 months of age	71.8	100.8	142.8	107.3	207.3
Add MenACWY at 3 and 12 months of age	79.8	113.7	157.2	118.9	230.2
Add MenACWY at 2, 4, and 12 months of age	86.9	125.2	169.8	129.1	250.3
Deaths Prevented (compared with base case of adolescent MenACWY immunisation alone)					
Add MenACWY at 3 months of age	10.5	15.1	20.4	15.5	30.2
Add MenACWY at 2 and 4 months of age	12.1	17.5	23.4	17.9	34.7
Add MenACWY at 12 months of age	6	7.6	12.8	9.2	17.5
Add MenACWY at 5 and 12 months of age	10.6	14.9	21.1	15.8	30.6
Add MenACWY at 3 and 12 months of age	11.8	16.8	23.3	17.6	34
Add MenACWY at 2, 4, and 12 months of age	12.8	18.5	25.1	19.1	37

## Data Availability

Data in this study are presented within the article and Supplementary Materials.

## Supplementary Materials

The following are available online at https://www.mdpi.com/article/10.3390/vaccines11050940/s1, Figure S1: IMD Model Structure, Table S1: IMD Vaccination Strategies Included in the Model [55], Table S2: Epidemiology Inputs [29,56], Table S3: Disease Manifestation Inputs, Table S4: Vaccine Efficacy Inputs: Waning and Herd Effect, Table S5: Case Fatality Probability Inputs, Table S6: Sequelae Inputs [57,58,59,60]; Table S7: Disutility Inputs Post-Acute IMD [61,62,63,64,65,66,67,68,69,70,71,72,73,74].
