# Supplementary material for: Assessing the Role of Infant and Toddler MenACWY Immunisation in the UK: Does the Adolescent MenACWY Programme Provide Sufficient Protection?
Source: Vaccines (Basel). 2023 May 4;11(5):940. doi: 10.3390/vaccines11050940 (PMC10220561; doi:10.3390/vaccines11050940)
Supplement: Supplementary file 1 [file vaccines-11-00940-s001.zip › vaccines-2249986 supplementary.pdf]

Supplementary Material

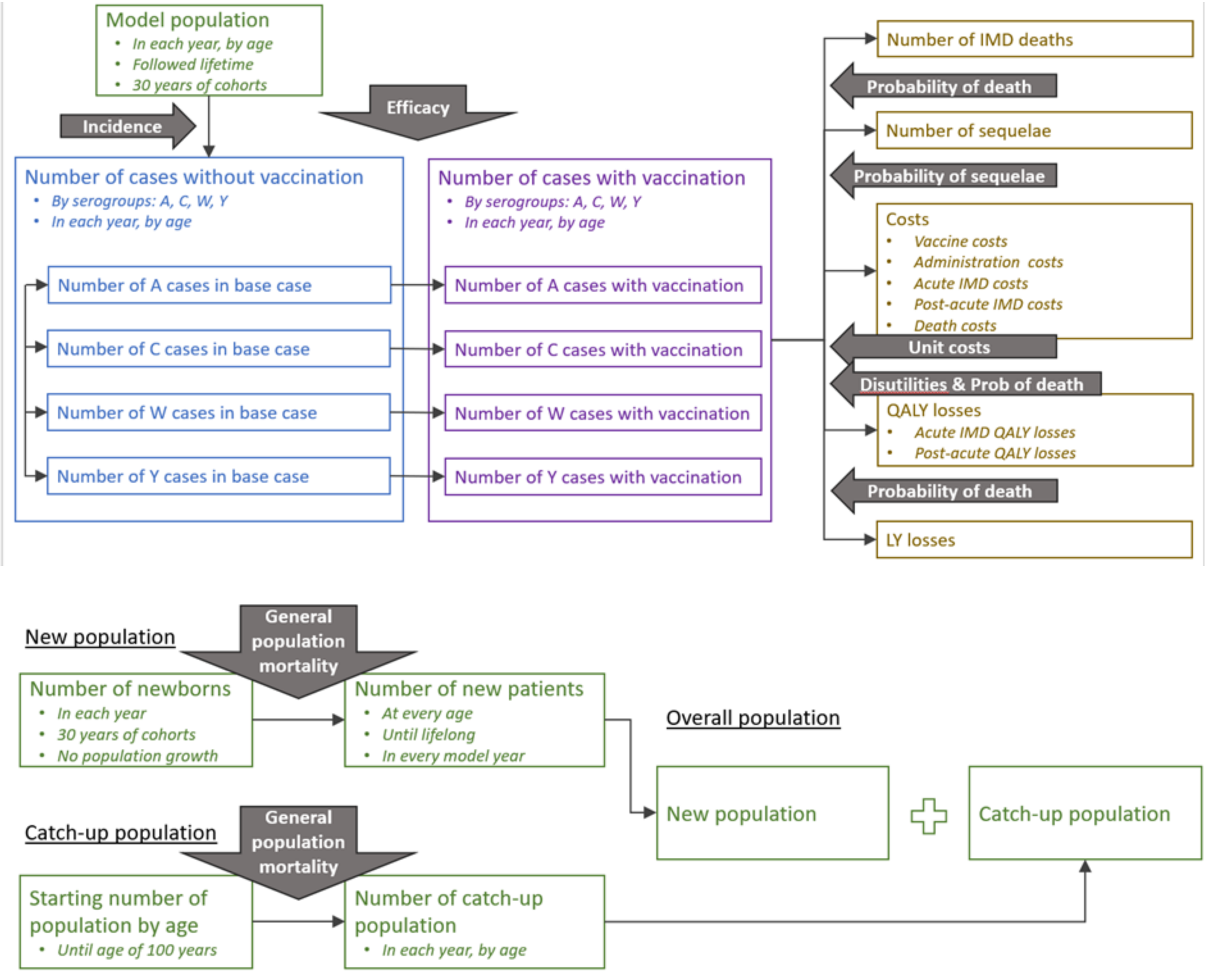

Figure S1. IMD Model Structure.

Table S1. IMD immunisation strategies included in model.

	**MenACWY (adolescent)**	**MenACWY (infant/toddler)**
**1**	14 years of age<br>Uptake: 86.2%	--
**2**	14 years of age<br>Uptake: 86.2%	3 months of age<br>Uptake: 96%
**3**	14 years of age<br>Uptake: 86.2%	2 & 4 months of age<br>Uptake: 96%, 89%, respectively
**4**	14 years of age<br>Uptake: 86.2%	12 months of age<br>Uptake: 96%
**5**	14 years of age<br>Uptake: 86.2%	5 & 12 months of age<br>Uptake: 96%, 89%, respectively
**6**	14 years of age<br>Uptake: 86.2%	3 & 12 months of age<br>Uptake: 96%, 89%, respectively
**7**	14 years of age<br>Uptake: 86.2%	2, 4 & 12 months of age<br>Uptake: 96%, 89%, 82% respectively

Note: Strategy 1 is the base case assumed future scenario where no Hib/MenC conjugate vaccine is available. Vaccine uptake is based on PHE 2020 [55].

Table S2. Epidemiology Inputs.

Age	Population size	IMD Incidence by Serogroup (per 100,000)			
		A	C	W	Y
age 0 - 12 months	722,881	0.00	0.15	1.12	0.62
age 1 - 2 years	752,554	0.00	0.07	0.24	0.08
age 2 - 5 years	2,381,828	0.00	0.07	0.24	0.08
age 5 - 10 years	4,149,852	0.00	0.05	0.03	0.05
age 10 - 15 years	3,953,866	0.00	0.05	0.03	0.05
age 15 - 23 years	6,107,639	0.00	0.04	0.14	0.15
age 23 - 25 years	1,702,409	0.00	0.04	0.14	0.15
age 25 - 45 years	21,828,709	0.00	0.04	0.02	0.03
age 45 - 65 years	12,822,108	0.00	0.04	0.06	0.10
age 65 - 100 years	12,374,961	0.00	0.04	0.17	0.26
Source:	2021 population data reported by the Office for National Statistics, Northern Ireland Statistics and Research Agency, National Records of Scotland, and Welsh Government [29]	Reported by the European Center for Disease Prevention and Control (ECDC), average 2005 to 2015 [56]			

Table S3. IMD Manifestation Inputs.

Manifestations	Clinical Manifestation Distribution by Serogroup			
	**A**	**C**	**W**	**Y**
Meningitis	42.9%	27.9%	16.2%	22.0%
Septicaemia	30.6%	44.2%	48.5%	41.6%
Meningitis and Septicaemia	20.4%	19.4%	15.6%	12.3%
Unspecified	6.1%	8.5%	19.7%	24.1%
Source:	Parikh et al. 2018 [5]			

Table S4. Vaccine Effectiveness Inputs: Waning and Herd Effect.

**Adolescent Herd Effect Assumption**	
**Incidence multiplier**	50%
**Source**	Carr 2022 [11]
**Waning Inputs**	
**Annual wanning rates after each dose**	
**Infant**	22.12%
**Toddler**	22.12%
**Adolescent**	5.85%
**Source**	Borja-Tabora 2020; Vesikari 2020 [24, 25]

Table S5. Case Fatality Probability Inputs.

Age	Case Fatality Rate
age 0 - 12 months	4.9%
age 1 - 2 years	4.8%
age 2 - 5 years	4.8%
age 5 - 10 years	4.7%
age 10 - 15 years	4.7%
age 15 - 23 years	6.8%
age 23 - 25 years	6.8%
age 25 - 45 years	7.1%
age 45 - 65 years	8.5%
age 65 - 100 years	22.8%
Source: Beebeejaun 2020 [33]	

Table S6. Sequelae Inputs.

Sequelae	Probability of Sequelae
Amputation	3.6%
Anxiety	2.7%
Arthritis	7.5%
Cognitive Impairment	0.0%
Depression	0.3%
Hearing Loss	6.3%
Migraine	0.6%
Motor Deficits	0.0%
Neurological Disability	3.1%
Renal Failure	0.3%
Seizure	2.6%
Skin Scarring	2.6%
Speech Problems	4.2%
Visual Impairment	0.4%
**Amputation**	**Distribution of Amputation**
Digital	71.5%
Single limb	14.2%
Multiple limb	14.2%
**Hearing Loss**	**Distribution of Hearing Loss**
Adaptive listening strategies	42.9%
Hearing aids	24.3%
Cochlear implants	32.8%
Source:	Amputation: Bettinger et al. 2013 [57], Hearing loss: Viner et al. 2012 [58], Amputations: Bettinger et al. 2013 [57], Anxiety: Viner et al. 2012 [58], Arthritis: Cabellos et al. 2012 [59], Cognitive Impairment: Assumed to be 0%, Depression: Bettinger et al. 2013 [57], Hearing Losses: Viner et al. 2012 [58], Migraine: Gottfredsson et al. 2011, Motor Deficits: Assumed to be 0%, Neurological Disability: Bettinger et al. 2013 [57], Renal Failure: Bettinger et al. 2013 [57], Seizure: Bettinger et al. 2013 [57], Skin Scarring: Rivero-Calle et al. 2016 [60], Speech Problems: Viner et al. 2012 [58], Visual Impairment: Viner et al. 2012 [58]

Note: duration of all sequelae is assumed to be lifelong.

Table S7. Disutility Inputs during Post-Acute IMD.

		Patient	Caregiver	Source
**Patient without sequelae**	**Disutility**	0.30	-	Koomen et al. 2005 [61]
	**Duration**	9 years	-	Schmand et al. 2010 [62]
**Caregivers after patient's IMD death**	**Disutility**	-	0.07	Song et al. 2010 [63]
	**Duration**	-	40 years	Assumption
**Sequelae disutilities**				
**Amputation: Digital**		0.194	0.110*	Gani et al. 2008 [64]
**Amputation: Single limb**		0.300		Shepard et al. 2005 [65]
**Amputation: Multiple limb**		0.310		Shepard et al. 2005 [65]
**Anxiety**		0.313		Saarni et al. 2007 [66]
**Arthritis**		0.310		Caban-Martinez et al. 2011 [67]
**Cognitive Impairment**		0.380		Donev, Measuing DALY [68]
**Depression**		0.210		Saarni et al. 2007 [66]
**Hearing loss: Adaptive listening strategies**		0.090		Oostenbrink et al. 2002 [69]
**Hearing loss: Hearing aids**		0.090		Oostenbrink et al. 2002 [69]
**Hearing loss: Cochlear implants**		0.190		Oostenbrink et al. 2002 [69]
**Migraine**		0.186		Xu et al. 2011 [70]
**Motor Deficits**		0.330		Stouthard et al. 1997 [71]
**Neurological Disability**		0.380		Shepard et al. 2005 [65]
**Renal Failure**		0.180		Wyld et al. 2012 [72]
**Seizure**		0.170		Oostenbrink et al. 2002 [69]
**Speech Problems**		0.220		Yfantopoulos et al. 2001 [73]
**Visual Impairment**		0.490		Brown et al. 2001 [74]
**Skin Scarring**		0.000	0.070*	Shepard et al. 2005 [65]

*Caregiver disutilities are assumed.
